# Pitaya Genome and Multiomics Database (PGMD): A Comprehensive and Integrative Resource of *Selenicereus undatus*

**DOI:** 10.3390/genes13050745

**Published:** 2022-04-24

**Authors:** Canbin Chen, Fangping Li, Fangfang Xie, Jiaxuan Chen, Qingzhu Hua, Jianye Chen, Zhijiang Wu, Zhike Zhang, Rong Zhang, Jietang Zhao, Guibing Hu, Yonghua Qin

**Affiliations:** 1 Guangdong Provincial Key Laboratory of Postharvest Science of Fruits and Vegetables/Key Laboratory of South China Horticultural Crop Biology and Germplasm Enhancement, Ministry of Agriculture, College of Horticulture, South China Agricultural University, Guangzhou 510642, China; nnchencanbin@163.com (C.C.); xiefangfang202012@163.com (F.X.); jxchen0127@163.com (J.C.); huaqingzhu@stu.scau.edu.cn (Q.H.); chenjianye@scau.edu.cn (J.C.); poloky2@163.com (Z.Z.); r-zhang@scau.edu.cn (R.Z.); zhaojietang@gmail.com (J.Z.); guibing@scau.edu.cn (G.H.); 2 Guangdong Provincial Key Laboratory of Plant Molecular Breeding, State Key Laboratory for Conservation and Utilization of Subtropical Agro-Bioresources, South China Agricultural University, Guangzhou 510642, China; lpf_bio@foxmail.com; 3 Horticultural Research Institute, Guangxi Academy of Agricultural Sciences, Nanning 530007, China; zhijiangwu@gxaas.net

**Keywords:** pitaya, comprehensive database, PGMD

## Abstract

Pitaya (*Selenicereus*) is a kind of novel fruit with a delicious taste and superior horticulture ornamental value. The potential economic impact of the pitaya lies in its diverse uses not only as agricultural produce and processed foods but also in industrial and medicinal products. It is also an excellent plant material for basic and applied biological research. A comprehensive database of pitaya would facilitate studies of pitaya and the other Cactaceae plant species. Here, we constructed pitaya genome and multiomics database, which is a collection of the most updated and high-quality pitaya genomic assemblies. The database contains various information such as genomic variation, gene expression, miRNA profiles, metabolite and proteomic data from various tissues and fruit developmental stages of different pitaya cultivars. In PGMD, we also uploaded videos on the flowering process and planting tutorials for practical usage of pitaya. Overall, these valuable data provided in the PGMD will significantly facilitate future studies on population genetics, molecular breeding and function research of pitaya.

## 1. Introduction

The pitaya, also known as pitahaya or dragon fruit, is a perennial climbing fruit crop belonging to the genus *Seleniereus* under the family Cactaceae [1]. Pitaya, originated from Central and South America, is one of the most famous fruits in tropical and subtropical areas. As a member of the Cactaceae, pitaya exhibits a range of specific adaptations to arid lands, such as succulent stems with spines instead of leaves, the crassulacean acid metabolism (CAM) pathway. It is becoming a popular fruit in Southeast Asia, China, Israel, Australia and Cyprus due to its attractive appearance, shocking fuchsia colors, delicious taste and high nutrients [2].

Multiomics methods such as whole-genome sequencing [3,4,5], transcriptomics [6,7], proteomics [8] and metabolomics [9] have been used to study the evolutionary process and physiological functions of pitaya. Recently, the genomic draft of the cultivar ‘David Bowie’ pitaya [3] and the high-quality chromosomal level genome of cultivar ‘Guanhuabai’ pitaya [4] were released. These data will greatly facilitate genome-wide studies of functional genes and understanding the evolution of pitaya. With increasing amounts of omics data available, a centralized platform is necessary for data storage and analyses of these large-scale datasets. The web-based public databases have been extensively characterized in plant species including *Cannabis sativa* L. [10], *Nelumbo nucifera* [11], *Lonicera japonica* [12], *Rhododendron* [13] and *Fragaria* spp. [14]. The development of web-based public databases will greatly benefit from the application of high-throughput sequencing and help researchers to study gene functions [10,11,12,13,14]. However, a web-based public database of pitaya is still unavailable, making it difficult to utilize the data comprehensively.

To meet the demands for pitaya genome and multiomics data resources, we established an integrated pitaya genome and multiomics database (PGMD; http://www.pitayagenomic.com (accessed on 20 April 2022)). The PGMD provides a comprehensive consultation service in terms of the information of the latest assemblies of pitaya genome, gene expression data, miRNA, proteome, metabolite and variation information among different pitaya cultivars, tissues and fruit developmental stages. By integrating the genomic, transcriptomic, proteomic and metabolomic data, PGMD can facilitate international collaboration and exchange of comprehensive and valuable information on basic and applied studies in pitaya. PGMD is also the first database of the cactus and will become an excellent central gateway to better understand the biology and genetics of Cactaceae. The release of PGMD will contribute to studies of genetic diversity and quality improvement in pitaya.

## 2. Data Records and Methods

### 2.1. Data Records

The raw sequence, assembly and annotation data of *Selenicereus undatus* (*S. undatus*) genome sequencing are deposited in the SRA (Sequence Read Archive) data resource of the NCBI with Bioproject ID PRJNA691451 [15] (Guanhuabai) and PRJNA664414 [16] (David Bowie). The transcriptome data of various developmental stages and tissues are deposited under PRJNA704510 [17] and PRJNA725049 [18]. The miRNA data are available in PRJNA588519 [19]. The Pitaya Genome Database website raw reads data have been submitted to Figshare database (10.6084/m9.figshare.16570611, accessed on 20 April 2022) [20]. The remaining unspecified data are released within the PGMD database.

### 2.2. Data Processing

Pipeline Hisat2-stringties-ballgown [21] was used for RNA-Seq quantity and FPKM data analyses. Software bwa mem (Version 0.7.17-r1188) (https://sourceforge.net/projects/bio-bwa/ (accessed on 20 April 2022)) and GATK were respectively used for Illumina short reads data alignment and variation detection [22,23]. For the genomic comparison of ‘Guanhuabai’ and ‘David Bowie’ pitayas, the programme Ragoo was used to rearrange and rescaffold the genome of the ‘David Bowie’ pitaya [24]. Alignment and the detection of structure variations were performed by minimap2 [25] and Syri software (Version v1.5.4) (https://schneebergerlab.github.io/syri/ (accessed on 20 April 2022)) [26]. The format conversion and sorting of intermediate files (.sam and .bam) were obtained by the programme Samtools (Version v1.9) [27].

### 2.3. Database Construction

All genomic sequence, annotation, gene expression, variation and miRNA data were stored via MySQL (Version 7.1.0) (https://www.mysql.com/ (accessed on 20 April 2022)) on a Centos7 server. A user-friendly website was developed using HTML5, JavaScript and PHP7, which can be accessed through different browsers, such as Google Chrome and Firefox. Gene models and transcript isoforms were provided via JBrowse [28,29]. Heatmaps, networks and histograms of gene, metabolites, protein expression and miRNA interaction relationships were plotted via network matplotlib, seaborn of python 3.8 and the module ECharts of JavaScript. The query searches were achieved via PHP7. Common utilities for genomic studies such as basic local alignment search tool (BLAST) and Sequence exactor were also deployed and accessible.

### 2.4. Code Availability

Genome and transcriptome sequence data were provided by the corresponding software and sequencing platform manufacturers (Illumina, San Diego, CA, USA and PacBio, Menlo Park, CA, USA). The software (including version, parameters, and setup) used for genome assembly and the detailed usage of the database are referenced in the sections of previous papers [3,4]. In the Figshare database [20], HTML5, JavaScript, Python, Perl and PHP code were uploaded to build the PGMD.

## 3. Results

### 3.1. Major Datasets

PGMD was constructed based on the pitaya genome and the corresponding multiomics data. For PGMD, two chromosomal level genomes were displayed: (i) ‘Guanhuabai’ pitaya genome constructed in our previous work is the most complete genome of pitaya species with 1.41 Gb size, ~127.15 Mb scaffold N50 and 0.5% missing rate [4]. (ii) ‘David Bowie’ pitaya published in previous research with a scaffold N50 = 109.7 Mb and assembly size = 1.33 Gb [3]. To further evaluate the quality of genome assemblies, benchmarking universal single-copy orthologs (BUSCO) analysis [30] was carried out. The number of 93.8% and 93.0% completely conserved eukaryotic genes were identified in the genomes of ‘Guanhuabai’ and ‘David Bowie’ pitayas, respectively [3,4]. The variation information of the other five pitaya cultivars, ‘Youcihuanglong’, ‘Dayeshuijing’, ‘Guihonglong’, ‘Guanghuahong’ and ‘SCAU-184’, were obtained based on the corresponding Illumina short-read sequencing data using the workflow GATK SNP [21]. The chromosome-level pitaya genome of cultivar ‘Guanhuabai’ was used as the reference genome in this process. The PGMD also covered 479.53 Gb gene expression profiles of transcriptome and miRNA data. These data were derived from pitaya flowers, peels and pulps. Moreover, gas chromatography–mass spectrometry (GC–MS) information of twelve metabolites (e.g., sugars and organic acids) from seven fruit development stages of four pitaya cultivars were furnished in PGMD for referring. Together with two mass spectrometry (MS)-based proteome data from two pulp developmental periods of ‘Guanhuahong’ pitaya (*S. monacanthus*), these datasets constituted the multiomics module of the PGMD. Additionally, to meet the requirement of agronomic cultivation and cultural popularization, we uploaded videos about pitaya flower opening processes and propagation methods in the video module.

### 3.2. Uses

**Figure 1:**
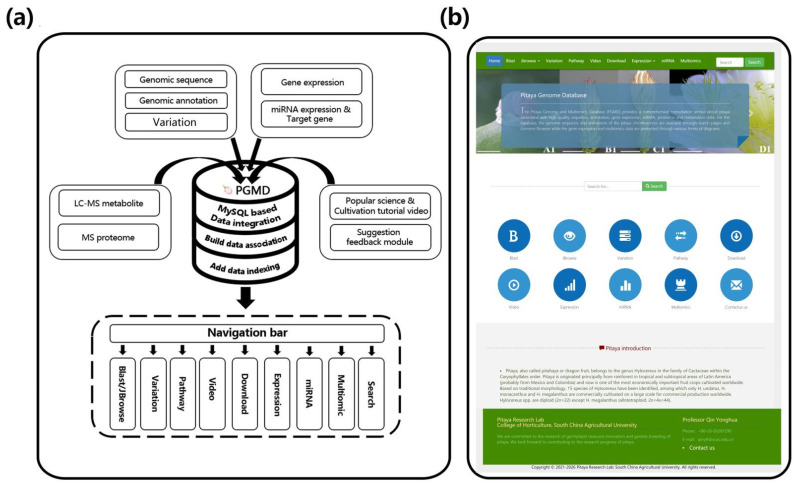
The flow diagram and home page of PGMD. (**a**) The flow diagram of PGMD. (**b**) The home page of PGMD.

**Figure 2:**
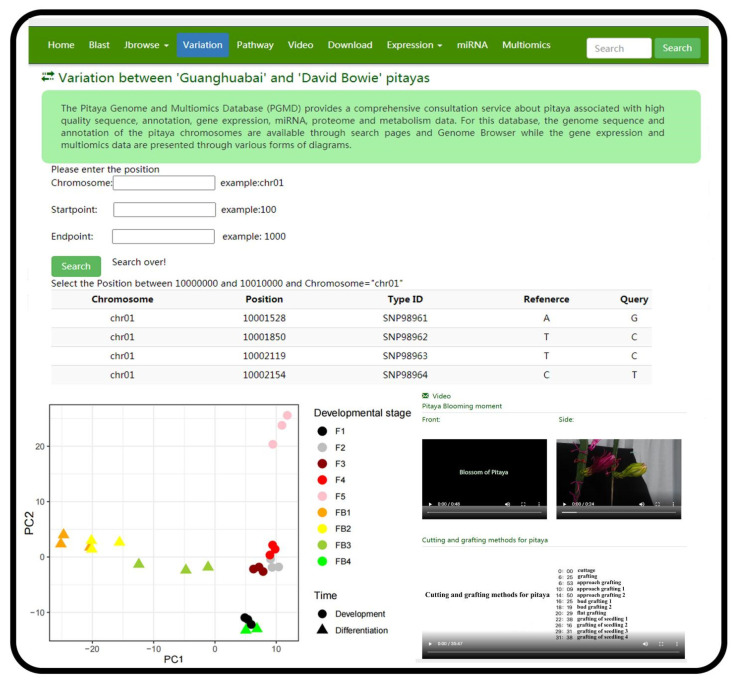
The screenshots of representative resources and action page examples for the ‘Variation’ and ‘Video’ modules. The ‘Variation’ and ‘Video’ module demonstrated genomic diversity among six pitaya cultivars, pitaya-related scientific periodicals and tutorial video; the PCA scatter diagram indicates the reproducibility and representativeness of the gene expression data.

**Figure 3:**
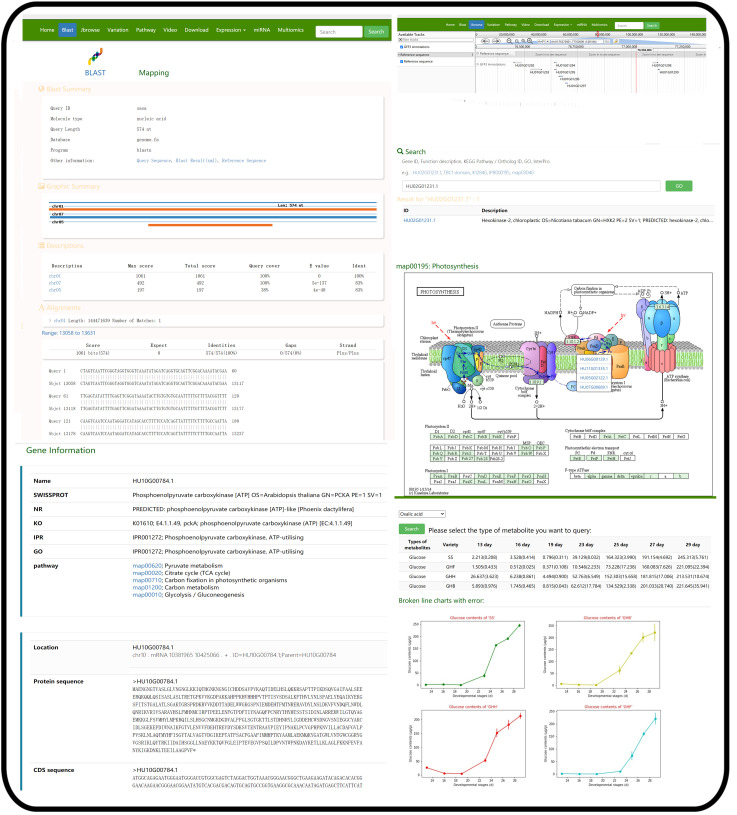
The screenshots of representative resources and action page examples for the ‘Blast’, ‘Gene Browse’ and ‘Search’ modules. The ‘Blast’, ‘Gene Browse’ and ‘Search’ modules showing detailed information of genes identified in this study including gene loci and sequence position of the gene in KEGG pathway. The trends of various metabolite data during pitaya fruit development were presented by line chart and table.

**Figure 4:**
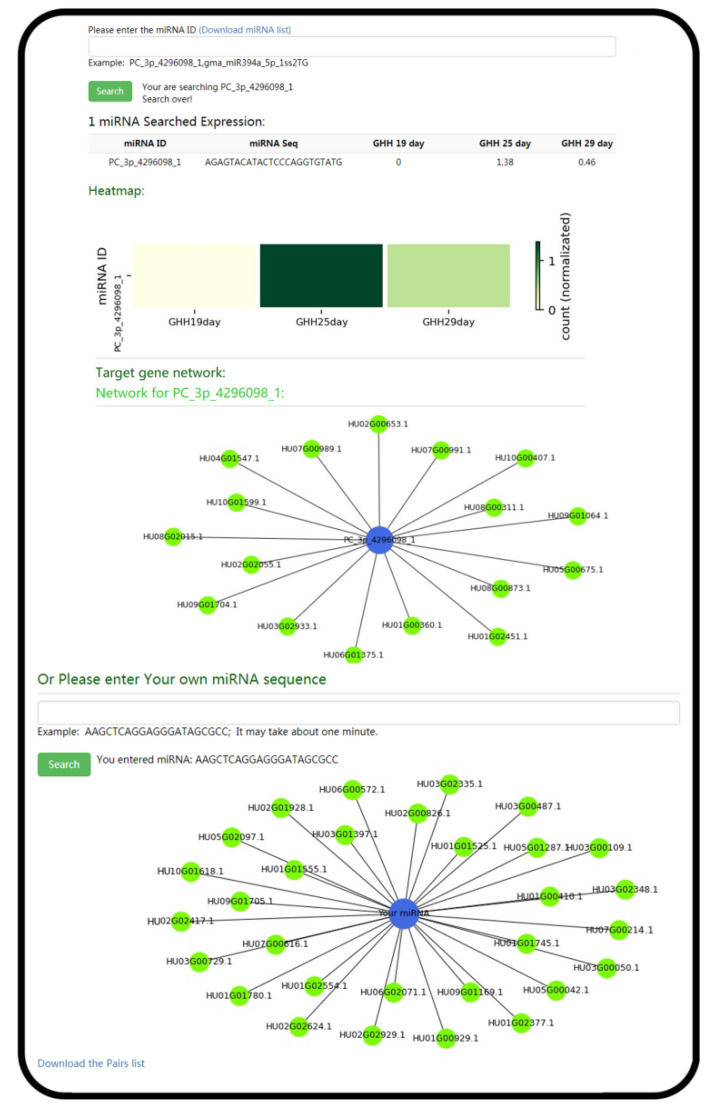
The screenshots of representative resources and action page examples for the ‘miRNA’ module. Expression levels of miRNA and corresponding target genes can be queried by ‘miRNA’ module, users can also perform prediction of target genes by entering miRNA sequences.

**Figure 5:**
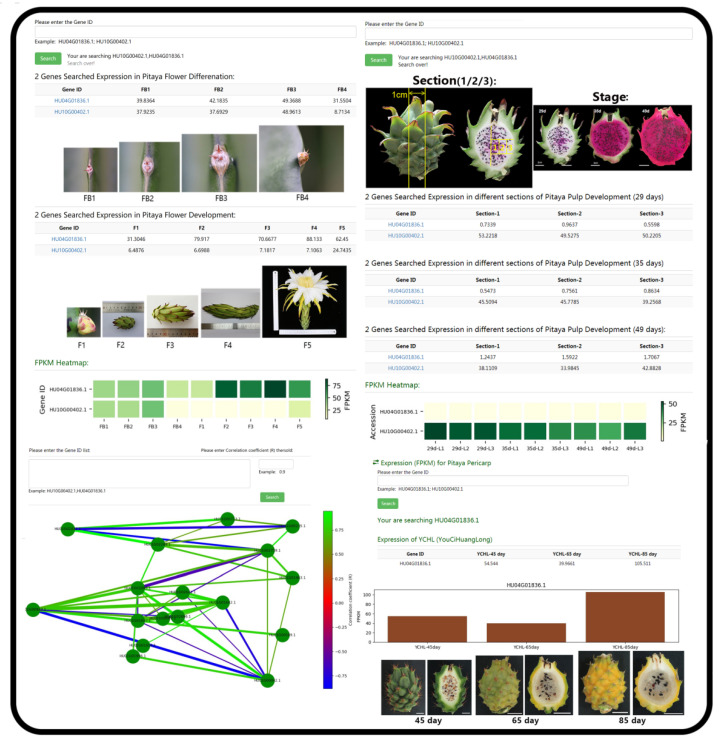
The screenshots of representative resources and action page example for the ‘Expression’ module. ‘Expression’ module displayed the gene expression data of each tissue in various development stages through the expression calorimetry, histogram and corresponding table forms. Users can input lists of gene symbols and gain the coexpression relationship and visual network diagram through the algorithm based on Pearson correlation coefficient in coexpression function of ‘Expression’ module.

For the utilization of PGMD, the navigation bar was used to access the functional pages (Figure 1a,b). The database had 10 modules. Among these modules, the ‘Variation’ module provided the information of variable sites of six pitaya cultivars based on the reference genome of ‘Guanhuabai’ pitaya. The data from the ‘David Bowie’ pitaya contained some large-scale structural variations (50 > bp), and the rest were SNP, short fragment insertion and deletion (Figure 2). The ‘Search’ option allows users to retrieve the gene information by inputting gene ID or pathway name (Figure 3). The ‘Jbrowse’ option provides a fast and interactive genome browser for navigating large-scale high throughput sequencing data under a genomic framework. This module also provides an interface for extracting the target sequence from a specified region. The pathway option provides a clear KEGG pathway maps-based functional annotation of pitaya genes and interactive visualization (Figure 3). The ‘BLAST’ option is capable of performing homology search with different datasets of pitaya genome (Figure 3). The ‘miRNA’ option furnishes a platform to access and visualize expression levels of known miRNAs and relevant target genes of pitaya by network diagrams and downloadable tables. This module also supports users in uploading their own miRNA sequences and perform target gene predictions (Figure 4). The ‘Download’ section allows users to freely obtain all data from PGMD in batches. The expression module provides expression data and corresponding visual charts of different pitaya tissues (flowers, pulps and peels) in various developmental stages (Figure 5). This module also furnishes analyses of gene coexpression. Users can input lists of gene symbols and gain coexpression relationships and visual network diagrams through the algorithm based on Pearson correlation coefficient (Figure 5). The ‘Multiomics’ module visualizes the GC-MS of various compounds and proteomic data through line charts and tables (Figure 3). Additionally, a ‘Contact us’ section enables users to communicate with us, which is necessary for the further improvement of PGMD.

### 3.3. Technical Validation and Data Visualization

Technical validation methods and steps were implemented in the construction of PGMD. Quality controls were performed to determine the reliability of data.

### 3.4. Genomic Data Validation

A high missing rate (the percentage of N exceeded 12%) of the genome file ‘David Bowie’ pitaya was detected [16], resulting in the considerable fragmentation and incompletion of the genome which prevented the further utilization. Therefore, we broke the scaffold level sequence of the genomic data and rearranged the contigs based on the homologous relationships from the more completed ‘Guanhuabai’ pitaya genome with the Ragoo workflow [24,26].

### 3.5. Gene Expression, miRNA and Multiomics Data Processing and Visualization

Principal component analysis (PCA analysis) of the gene expression data across different RNA-Seq (FPKM) and miRNA samples (read counts) was performed to ensure the representativeness and accuracy using R package DESeq2 [31]. Most of biological duplicates of the same site, developmental stage and genetic background were well clustered, which elucidated prominent representativeness and accuracy of these samples (Figure 2). Median values in these biological repetitions were implemented for presentation and plotting in PGMD. Additionally, GC-MS data of 12 metabolites were obtained according to the comparison of peak positions with standard samples. The mean values of at least three biological duplicates with acceptable standard error were displayed by broken line graphs with corresponding phenotype pictures.

## 4. Discussion and Conclusions

Release of a chromosome-scale genome sequence of pitaya (*S. undatus*) data provides a global view of the regulatory network of betalain biosynthesis in pitaya [4]. However, no comprehensive database for gene functional analysis in *Selenicereus* has been established. PGMD is dedicated to providing a comprehensive database of *Selenicereus* multiomics data. The current implementation of PGMD integrates important data, including various information of genomic variation, gene expression, miRNA profiles, metabolite and proteomic data, from various tissues and fruit developmental stages of different pitaya cultivars. It also provides a series of tools for online data analysis and visualization. The PGMD is good for resource sharing, research funds saving and gene screening. To allow further exploration of the molecular mechanisms involved in the betalain biosynthesis of pitaya, we will continue to update the datasets when new data are obtained. For instance, our team will release the genome of *S. monacanthus* and its phenotypic datasets, including transcriptomic and metabolite data, in the near future. The database will provide valuable information for molecular study of *Selenicereus*.

## Data Availability

Data is contained within the article and figshare.
